# A Comparison of Experimental and Analytical Procedures to Measure Passive Drag in Human Swimming

**DOI:** 10.1371/journal.pone.0130868

**Published:** 2015-07-24

**Authors:** Tiago M. Barbosa, Jorge E. Morais, Pedro Forte, Henrique Neiva, Nuno D. Garrido, Daniel A. Marinho

**Affiliations:** 1 National Institute of Education, Nanyang Technological University, Singapore, Singapore; 2 Department of Sport Sciences, Polytechnic Institute of Bragança, Bragança, Portugal; 3 Department of Sport Sciences, University of Beira Interior, Covilhã, Portugal; 4 Department of Sport Sciences, University of Trás-os-Montes and Alto Douro, Vila Real, Portugal; 5 Research Centre in Sports, Health and Human Development, Vila Real, Portugal; University of California San Diego, UNITED STATES

## Abstract

The aim of this study was to compare the swimming hydrodynamics assessed with experimental and analytical procedures, as well as, to learn about the relative contributions of the friction drag and pressure drag to total passive drag. Sixty young talented swimmers (30 boys and 30 girls with 13.59±0.77 and 12.61±0.07 years-old, respectively) were assessed. Passive drag was assessed with inverse dynamics of the gliding decay speed. The theoretical modeling included a set of analytical procedures based on naval architecture adapted to human swimming. Linear regression models between experimental and analytical procedures showed a high correlation for both passive drag (D_p_ = 0.777*D_f+pr_; R^2^ = 0.90; R^2^_a_ = 0.90; SEE = 8.528; P<0.001) and passive drag coefficient (C_Dp_ = 1.918*C_Df+pr_; R^2^ = 0.96; R^2^_a_ = 0.96; SEE = 0.029; P<0.001). On average the difference between methods was -7.002N (95%CI: -40.480; 26.475) for the passive drag and 0.127 (95%CI: 0.007; 0.247) for the passive drag coefficient. The partial contribution of friction drag and pressure drag to total passive drag was 14.12±9.33% and 85.88±9.33%, respectively. As a conclusion, there is a strong relationship between the passive drag and passive drag coefficient assessed with experimental and analytical procedures. The analytical method is a novel, feasible and valid way to gather insight about one’s passive drag during training and competition. Analytical methods can be selected not only to perform race analysis during official competitions but also to monitor the swimmer’s status on regular basis during training sessions without disrupting or time-consuming procedures.

## Introduction

Human swimming is a major topic of research for biomechanists and sport scientists. Water, being such a challenging and “unnatural" environment for humans, makes this a very exciting research topic. In addition, competitive swimming is one of the most popular sports around the world and the second Olympic sport after track and field. Therefore, swimmers, coaches, and sports analysts are also keen to have deeper insights about the determinant factors that may affect their performances.

Swimming is a multifactorial phenomenon where several fields play a role, albeit biomechanics and physiology are on the top of the list [1]. The swimmer’s hydrodynamic profile is one of the main biomechanical factors that determine performance. The core of a hydrodynamic analysis is the measurement of the drag force [2]. For a long time, researchers put much effort to develop and validate procedures to measure or estimate the drag force acting upon a swimmer [3,4]. The drag force is an external force applied to the swimmer and has a direction opposite to his displacement:

$$D=\frac{1}{2}\cdot \rho \cdot v^{2}\cdot S\cdot C_{D}$$ (1)

where *D* is the drag force, *ρ* is the fluid’s density, *v* is the velocity, and *S* is the projected frontal surface area. The forward displacement (derived by the velocity or the acceleration) is the resultant of the external forces acting on the swimmer’s body:

$$a=\frac{\sum F_{i}}{m}$$ (2)

where *a* is the acceleration, Σ*F*_*i*_ is the resultant force, and *m* is the body mass. Eq 2 can be changed to the following:

$$a=\frac{F_{P}+(-D)}{m}$$ (3)

where *a* is the acceleration, *F*_*P*_ is the total propulsive force, *D* is the drag force, and *m* is the body mass. Hence, based on Eq 3, it is easy to follow that the swimmer’s performance depends on inertial characteristics (i.e., body mass), thrust (i.e., propulsion), and resistance (i.e., drag force). The drag force is termed “passive drag” when acting upon a swimmer that is towed or gliding, without any limb actions [5]. Active drag is the name for the force when applied to a swimmer performing limb action to propel himself in water [5]. There is anecdotal and empirical evidence that swimmers stay for a longer period gliding during a race compared with previous recommendations in the literature (e.g., after the start, turns, and in some events, such as breaststroke between stroke cycles) [6,7]. During these phases the swimmer is passive, thus passive drag becomes an important parameter to be monitored during training and competition.

To learn about one’s passive drag, literature reports a few: (i) experimental tests (i.e., towing and gliding tests or inverse dynamics of the velocity gliding decay), and (ii) numerical simulations (i.e., computer fluid dynamics). Over experimental testing, the subject held onto a handle that is attached by a cable through pulleys to a dynamometer, and the force produced towing him at a constant speed is measured [8]. Alternatively, drag force can be estimated based on the inverse dynamics of the velocity gliding decay after a maximal push-off [2]. This procedure is similar to the cost-down testing to measure drag force on land-based settings [9]. The method is based on fitting a velocity function to the derived velocity data over time and thereafter run the inverse dynamics. Others suggested a technique (coined as “hydro-kinematic method”) based on the fitting of the displacement function to the displacement data, enabling to measure the glide efficiency as well [10]. Numerical simulations are run after scanning the swimmer, inputting his anthropometrical features plus boundary conditions and setting the equations that govern the motion [11]. Both experimental testing and numerical simulations are expensive, time consuming, needing trained and dedicated researchers or technicians. On top of that, they are disruptive of the training sessions and unfeasible during official events. However, researchers and practitioners are keen to have a deeper insight about a swimmer hydrodynamics in realistic settings, during official competitions and on a daily basis during training sessions.

In naval architecture, engineers can analyze the vessel’s hydrodynamics based on experimental testing, numerical simulations, and analytical procedures [12]. There are a set of mathematical models that can be used to have some insight about a vessel hydrodynamics (e.g., sailing, canoeing, kayaking, surfing, rowing). Recently, that set of mathematical models was adapted to human swimming [13]. However, it remains to be shown how valid and reliable these procedures are. If such procedures can be adapted to human swimming and validated with one of the mainstream procedures reported in the literature, this would be a true breakthrough and has a positive impact on the evidence-based practice of coaches and sport analysts.

An added value of the analytical procedures is that they also enable us to learn about the partial contribution of each drag component to total drag:

$$D=D_{f}+D_{pr}+D_{w}$$ (4)

where *D* is the total drag force, *D*_*f*_ is the friction drag, *D*_*pr*_ is the pressure drag, and *D*_*w*_ is the wave drag. *D*_*f*_ is due to the interaction between the fluid’s viscosity and the body’s surface. *D*_*pr*_ is related to the differences between pressure at the leading and trailing edges of the body (i.e., separation of the boundary layer with its attendant vortices). *D*_*w*_ reflects the energy needed to push the water out of the way of the body (i.e., the body is used to lift the water against gravity leading to the formation of waves). The *D*_*w*_ is significant if one is displacing on or close to the water surface. The *D*_*w*_ is negligible at depths greater than 1.8 chest depths below the surface [14]. Numerical simulations suggest that *D*_*pr-*_ *D*_*f*_ contributions are 85% to 15% [15] or 75% to 25% [16] of the total drag force of a gliding swimmer fully immersed in the prone position. However, quantifying the *D*_*pr-*_ *D*_*f*_ contributions to the total drag with other procedures besides numerical simulations has been extremely difficult [10]; and reported as being 65% to 35% wearing a waist-knee swimsuit and 71% to 29% in a conventional suit [8].

The aim of this paper was to compare the swimming hydrodynamic of humans collected with experimental and analytical procedures. Furthermore, to learn about the partial contribution of the friction and pressure drag to total drag. It was hypothesized that, although there is a strong relation between both procedures, a difference should exist and hence needs a correction factor. *D*_*pr*_ would play a major role in the total drag force in comparison to the *D*_*f*_.

## Materials and Methods

### Subjects

Sixty young swimmers (30 boys and 30 girls with 13.59 ± 0.77 and 12.61 ± 0.07 years old, 67.33 ± 6.24, and 72.50 ± 6.10 s of personal best at the 100-m freestyle event, respectively; all in Tanner stages 2–3 by self-report) were assessed. The sample included several age group national record holders; age-group national champions; and other swimmers that are part of a national talent identification, development, and follow-up scheme.

Written consent by parents or guardians and written assent by the underage swimmers were provided to take part in this study. All procedures were in accordance to the Helsinki Declaration regarding human research. The University of Trás-os-Montes and Alto Douro Ethic Committee also approved the study design (ethic review: UTAD-2011-219).

### Procedures

#### Anthropometrics and inertial parameters

Body mass (BM) was measured with a digital weight scale (SECA, 884, Hamburg, Germany) (ICC = 0.99). The swimmers’ added water mass (m_a_) was estimated as being approximately 27% (26.8 ± 2.3%, mean ± SE) for subjects with similar age to this study [17]. Height was measured with a digital stadiometer (SECA, 242, Hamburg, Germany) in the upright position, barefoot, and in swimwear (ICC = 0.99). All anthropometric measurements were collected according to standardized procedures. The wetted surface area of the body was determined by the Du Bois formula as follows [13]:

$$A_{wetted}=0.20247\cdot H^{0.725}\cdot BM^{0.425}$$ (5)

where *H* is the subject´s body height (in m) and *BM* is the body mass (in kg).

The trunk transverse surface area (S) was measured with a photogrammetric technique [18]. Swimmers were photographed with a digital camera (DSC-T7; Sony, Tokyo, Japan) in the transverse plane from above. Subjects stood on land, in the upright and streamlined position (i.e. length of the swimmer, L). This position is characterized by having the arms fully extended above the head, one hand over the other, fingers also extended close together, and head in neutral position. Subjects wore a regular textile swimsuit, cap, and goggles. On the camera-shooting field, a calibration frame with 0.945 m length was aside the swimmer at the shoulder level. The *S* was measured with an area-measuring software (UD Ruler, AVPSoft, USA) after importing the digital picture (ICC = 0.97).

#### Experimental procedure

The passive drag was assessed with inverse dynamics of the gliding decay speed [19]. This inverse dynamic technique aims to determine the forces that must act to resist a given motion (in this case, the drag force during streamlined gliding). Drag force is estimated from kinematic and inertial properties. Just like in mainstream inverse dynamics, differentiation of the speed-time set or double differentiation of the displacement-time sets is done and thereafter inserted in the basic equations of motion. This procedure has been reported on a regular basis in the literature [2,20,21]. Swimmers were invited to perform a maximal push-off on the wall fully immersed, at a self-selected depth, ranging approximately between 0.5 and 1.0 m to avoid *D*_*w*_ [22]. They were instructed to perform the glides in a streamlined position (head in neutral position, looking at the bottom of the swimming pool, legs fully extended and close together, arms fully extended at the front, and with one hand over the other) with no limb actions. Testing ended when swimmers broke the surface and/or were not able to make any further horizontal displacement gliding and/or started any limbs’ actions.

A speedometer cable (Swim speedometer; Swimsportec, Hildesheim, Germany) was attached to the swimmer’s hip, and the gliding velocity decay was acquired online (*f* = 50Hz) [21]. Data were exported to a signal-processing software (AcqKnowledge v. 3.9.0; Biopac Systems, Santa Barbara, USA) and filtered with a 4-Hz cutoff low-pass 4th order Butterworth filter. The selection of the cut-off value was done according to residual analysis (residual error versus cut-off frequency).

The gliding mean velocity and the corresponding mean acceleration based on the acceleration to time were calculated with moving time frame windows. The acceleration to time curve was obtained by numerical differentiation of the filtered speed-time curve, using the 5th order-centered equation [20]:

$$a_{i}\;=\;\frac{2v_{i-2}-16v_{i-1}+16v_{i+1}-2v_{i+2}}{24\Delta t}$$ (6)

where *a*_*i*_ is the hip’s instantaneous acceleration, *v*_*i*_ is the hip’s instantaneous velocity, and *t* is the time. Passive drag (D_p_) was calculated as follows:

$$D_{p}=\left(BM+m_{a}\right)\cdot a$$ (7)

where *D*_*p*_ is the passive drag, *BM* is the body mass, *m*_*a*_ is the added water mass, and *a* is the acceleration. The passive drag coefficient (C_Dp_) was calculated as follows:

$$C_{Dp}=\frac{2\cdot D_{p}}{\rho \cdot S\cdot v^{2}}$$ (8)

where *ρ* is the density of the water (being 1000 kg/m^3^), *D*_*p*_ is the passive drag, *v* is the gliding velocity, and *S* is the projected frontal surface area.

#### Analytical procedure

Theoretical modeling included a set of analytical procedures based on naval architecture but adapted to human swimming [13]. As the subjects were fully immersed, it was assumed that total drag is the sum of the friction and pressure components. The friction drag coefficient was determined from the ITTC-57 correlation line for turbulent flow [23]:

$$C_{Df}=\frac{0.075}{{\left(log(Re)-2\right)}^{2}}$$ (9)

where *Re* is the Reynolds number of the body and calculated as follows:

$$\text{Re}=\frac{v\cdot L}{\upsilon }$$ (10)

where *v* is the gliding velocity collected with the speedometer, *L* is the body length, and *ν* is the water kinematic viscosity (being 8.97 × 10^−7^ m^2^/s at 26°C). The friction drag (D_f_) was computed as follows:

$$D_{f}=0.5\cdot \rho \cdot v^{2}\cdot A_{wetted}\cdot C_{Df}$$ (11)

where *ρ* is the density of the water (being 1000 kg/m^3^), *v* is the gliding velocity collected with the speedometer, *A*_*wetted*_ is the wetted surface area, and *C*_*Df*_ is the friction drag coefficient. Pressure drag (D_pr_) was assumed to be due to bluff body separation, with viscous pressure resistance due to negligible boundary layer growth [13]:

$$D_{pr}=0.5\cdot \rho \cdot v^{2}\cdot S\cdot C_{Dpr}$$ (12)

where *ρ* is the density of the water (being 1000 kg/m^3^), *v* is the gliding velocity collected with the speedometer, *S* is the trunk transverse surface area, and *C*_*Dpr*_ is the pressure drag coefficient. The *C*_*Dpr*_ (0.23) was retrieved from numerical simulations in human swimming reported in the literature, hence adjusted to the human body geometry, glide depth and, for what it’s worth, the range of speeds performed over the glides [15,24]. Total passive drag was estimated as being the sum of *D*_*f*_ and D_pr_:

$$D_{f+pr}=D_{f}+D_{pr}$$ (13)

Total drag coefficient (*C*_*Df+pr*_) was computed thereafter inputting the *A*_*wetted*_.

### Statistical Analyses

Sample power was calculated for an α error probability of 0.05, a slope of 0.15, and a power (1-β) of 0.95 for simple linear regression models (one group, size of the slope), suggesting a total sample size of at least 54 subjects (GPower, v.3.1.7, University of Kiel, Germany). As it was challenging to recruit such number of adult/elite swimmers, it was decided to recruit young talented counterparts. The pool of young talented swimmers is greater, and they are highly skilled and proficient.

Minimum, maximum, mean, one standard deviation, and coefficient of variation are reported for all variables. Partial contribution (i.e., percentage) of pressure drag and friction drag to total drag is also reported but only for the analytical procedure. To date, experimental approaches have not broken down the total drag into friction and pressure components.

Simple linear regression models between assessed (experimental procedure) and estimated (analytical procedure) were computed for absolute values and after logarithmic transformation. Trendline equation, determination coefficient (R^2^), adjusted determination coefficient (R_a_^2^), and standard error of estimation (SEE) were calculated. As a rule of thumb, for qualitative and effect size analysis, it was defined that the relation was as follows: (i) very weak if R^2^ < 0.04, weak if 0.04 ≤ R^2^ < 0.16, moderate if 0.16 ≤ R^2^ < 0.49, high if 0.49 ≤ R^2^ < 0.81, and very high if 0.81 ≤ R^2^ < 1.0. Scattergrams include the main trendline plus the 95% confidence interval limits.

Bland-Altman analysis included the plot of the mean value versus the difference between experimental and analytical procedures. The limits of agreement were set at a ±1.96 standard deviation of the difference (i.e., 95% confidence interval). For qualitative interpretation, it was assumed that estimated data were valid if at least 80% of the plots were within the 95% confidence interval limits [18].

## Results

We failed to find significant differences by gender (D_p_: P = 0.25, d = 0.30; D_f+pr_: P = 0.45, d = 0.17; C_Dp_: P = 0.14, d = 0.39: C_f+pr_: P = 0.37, d = 0.23). Both genders seem to be completely blended on each other’s. Each gender shows a trendline similar to the one reported for the pooled sample. Therefore, data is reported only for the overall sample.

Coefficients of variation ranged between 0.04 and 0.18 for anthropometrical and inertial parameters and between 0.34 and 0.56 for the hydrodynamic variables (Table 1). Hence, variability is higher for the hydrodynamic profile than the anthropometric traits. The *D*_*p*_ was 48.92 ± 17.88N, and the *D*_*f+pr*_ was 57.14 ± 32.46N. The *C*_*Dp*_ was 0.263 ± 0.098, whereas the *C*_*Df+pr*_ was 0.137 ± 0.047.

**Table 1:** Descriptive statistics for the selected anthropometric, inertial and hydrodynamic variables.

	BM [kg]	m_a_ [kg]	H [m]	L [m]	S [cm^2^]	Re [dimens.]	A_wetted_ [m^2^]	D_p_ [N]	D_f+pr_ [N]	C_Dp_ [dimens.]	C_Df+pr_[dimens.]
Mean	51.58	13.93	1.62	1.89	701.4	2.53x10^6^	1.53	48.92	57.14	0.263	0.137
1 SD	8.47	2.29	0.08	0.10	128.1	3.35x10^5^	0.16	17.88	32.46	0.098	0.047
Minimum	37.80	10.21	1.49	1.75	460.3	1.81x10^6^	1.27	12.88	9.48	0.050	0.040
Maximum	73.20	19.76	1.81	2.11	983.5	3.43x10^6^	1.92	88.10	130.71	0.452	0.261
CV	0.16	0.16	0.04	0.05	0.18	0.13	0.10	0.36	0.56	0.37	0.34

BM—body mass; m_a_—added water mass, H—height; L—body length in the upright and streamlined position; S—trunk transverse surface area; Re—Reynold number; A_wetted_—wetted surface area; D_p_—passive drag assessed with the experimental procedure; D_f+pr_—passive drag assessed with the analytical procedure; C_Dp_—passive drag coefficient assessed with the experimental procedure; C_Df+pr_—passive drag coefficient assessed with the analytical procedure; dimens.—dimensionless.

**Fig 1:**
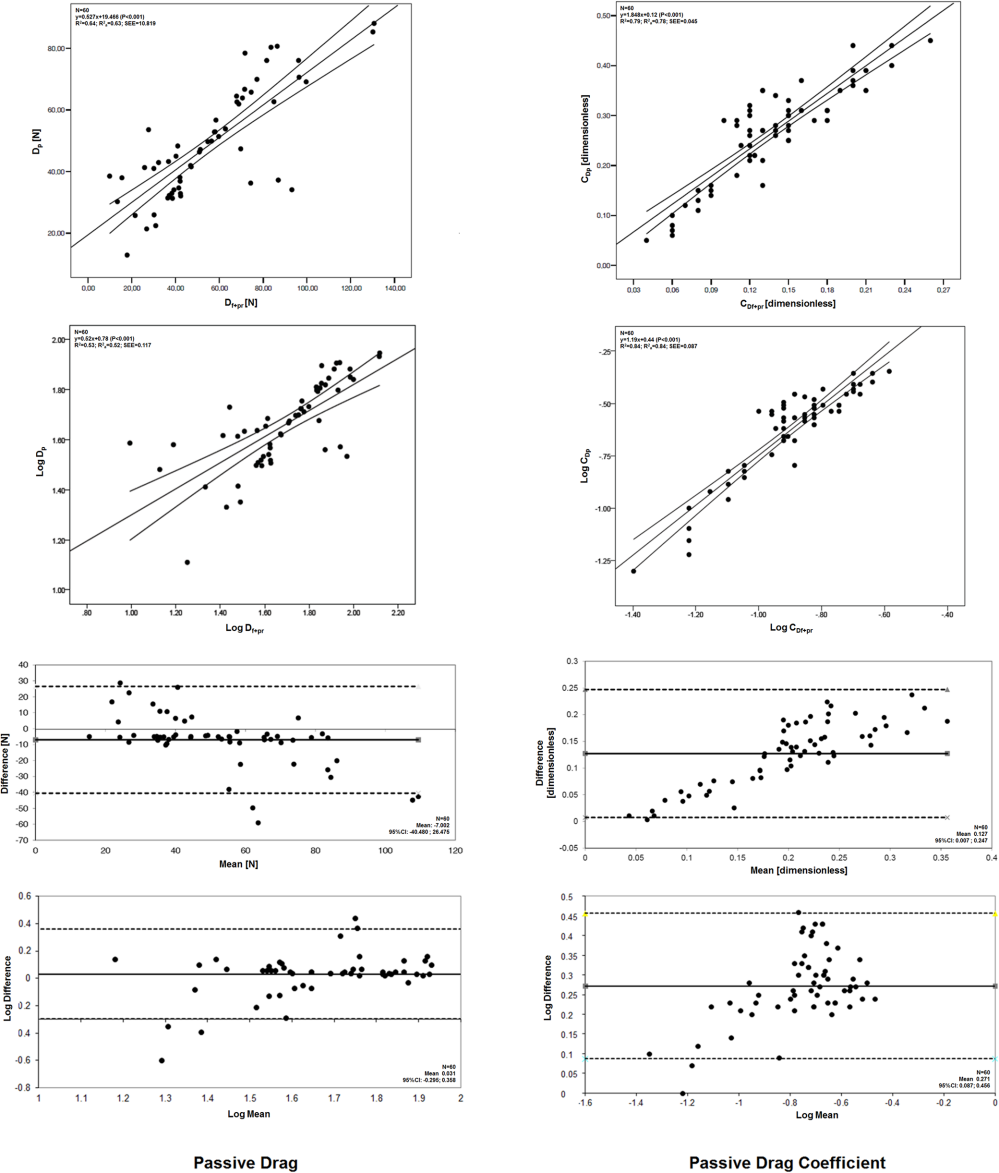
Comparison between experimental and analytical procedures to assess the passive drag (D_p_ and D_f+pr_, respectively) and the passive drag coefficient (CD_p_ and CD_f+pr_, respectively) in absolute unit and after logarithmic transformation.

Linear regression models between experimental and analytical procedures showed a high adjustment for both passive drag (absolute values: R^2^ = 0.64, R^2^_a_ = 0.63, SEE = 16.436, P < 0.001; log-log: R^2^ = 0.53, R^2^_a_ = 0.53, SEE = 0.117, P < 0.001) and passive drag coefficient (absolute values: R^2^ = 0.79, R^2^_a_ = 0.78, SEE = 0.021, P < 0.001; log-log: R^2^ = 0.84, R^2^_a_ = 0.84, SEE = 0.087, P < 0.001) (Fig 1). Visual inspection of the two scattergrams helps to understand that the 95% confidence intervals are quite narrow. If the subject does not glide forward in water (i.e., v = 0 m/s), there will be no drag force acting upon his body (D = 0N, Eq 1). Thus, when the models were forced to have the trendline crossing the origin (i.e., c = 0, so y = m^.^x), its adjustment increases furthermore for both passive drag (R^2^ = 0.90, R^2^_a_ = 0.90, SEE = 8.528, P < 0.001) and passive drag coefficient (R^2^ = 0.96, R^2^_a_ = 0.96, SEE = 0.029, P < 0.001):

$$D_{p}=0.777\cdot D_{f+pr}$$ (14)

$$C_{Dp}=1.918\cdot C_{Df+pr}$$ (15)

According to the Bland-Altman analysis, on average, the difference for the passive drag was -7.002N (95% CI: -40.480 to 26.475) having 48 of the 60 subjects showed an underestimation. Ten subject showed clear underestimations (difference higher than -20N). Remaining ones are within the (-10;0N) band (n = 38) or have an overestimation (n = 12). In this context a 10N difference can be considered a marginal bias. Hence 50 subjects are overestimations or neutrals (i.e. with a residual difference between procedures). The passive drag coefficient had a difference between techniques of 0.127 (95% CI: 0.007 to 0.247) and 59 of the 60 subjects showed an overestimation. Regarding logarithmic transformation, the difference for the passive drag was 0.031 (95% CI: -0.295 to 0.358) and 0.271 for the passive drag coefficient (95% CI: 0.087 to 0.456). The model shows a better adjustment in absolute values for the passive drag and in log-log transformation for the drag coefficient. Visual inspection of the plots revealed that the vast majority are within the 95% limits of agreement. Hence, based on data reported in Fig 1 plus Eqs 14 and 15, the bias between experimental and analytical procedures can be corrected whenever suitable.

**Fig 2:**
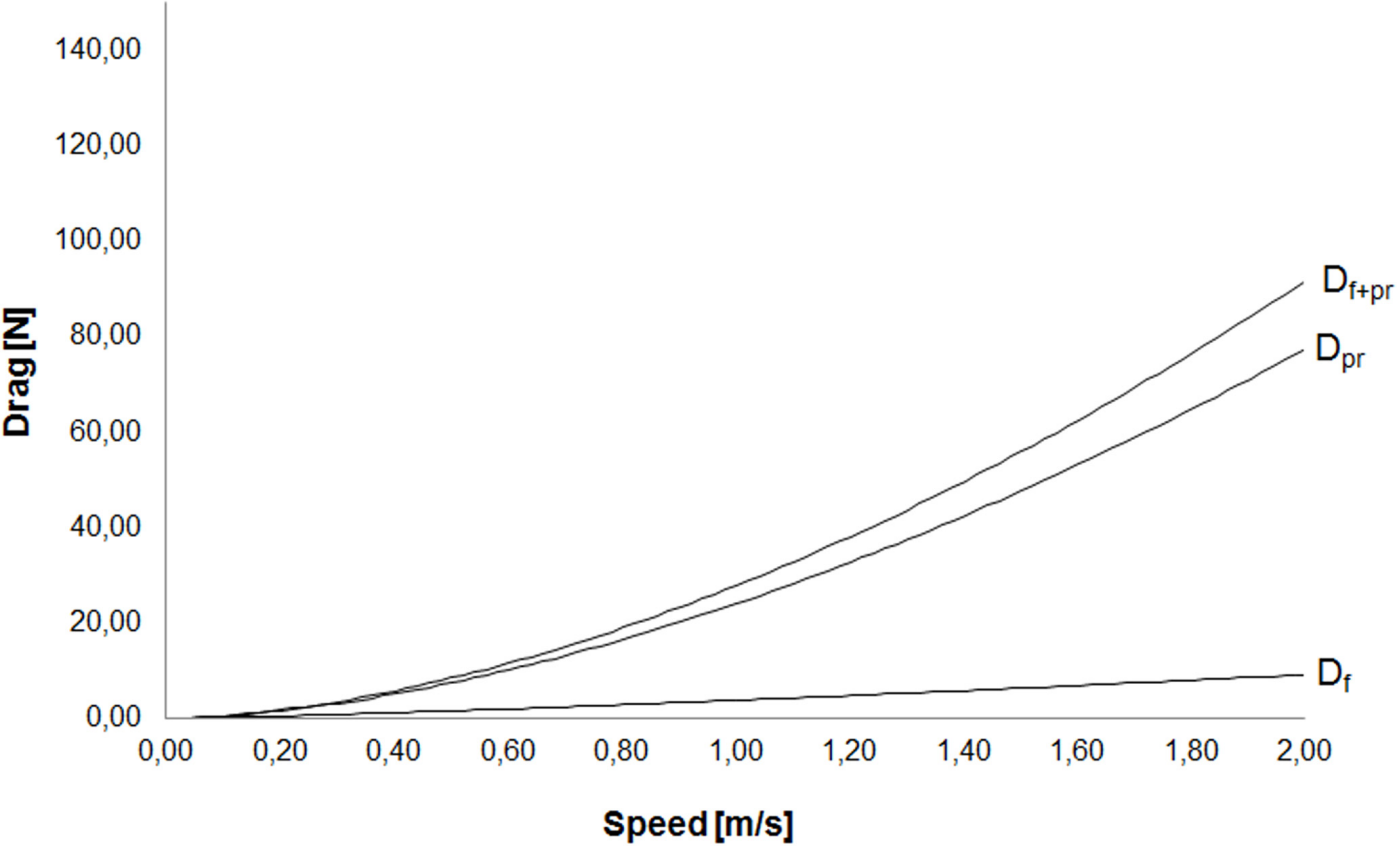
Breakdown of total passive drag (D_f+pr_, Eq 13) into friction drag (D_f_, Eq 11) and pressure drag (D_pr_, Eq 12) gliding fully immersed.

Regarding the analytical procedures, the partial contribution of *D*_*f*_ and *D*_*pr*_ to total passive drag was 14.12 ± 9.33% and 85.88 ± 9.33%, respectively. Therefore, *D*_*pr*_ is the major determinant of both components to total drag (Fig 2).

## Discussion

Our aim was to compare the swimming hydrodynamics assessed with experimental and analytical procedures and to learn about the *D*_*pr-*_ *D*_*f*_ contribution to total passive drag. There is a very strong relation between both procedures for the passive drag and passive drag coefficient. The partial contribution of *D*_*pr-*_ *D*_*f*_ contribution to total drag is roughly 85% to 15%.

The ITTC-57 correlation model (Eq 9) was proposed by R. N. Newton at the 1957 Meeting of the International Towing Tank Commission (ITTC) held in Madrid. It is the most popular method in naval architecture to estimate the friction drag, probably because it agrees with Schoenherr line at Re > 10^7^. However, it is common to be selected for small ship models (i.e., lower Re; e.g., Re~10^6^ that is within the ranges shown by our swimmers). This same approximation was reported at least in an earlier study on human swimming by another research group [13]. In the literature, we can also find several sets of correlations to estimate the friction drag coefficient. Most approximation formulas were computed for a turbulent boundary layer on a flat plate. We compared the ITTC-57 with some of the approximations that are suggested as being more accurate for the range 10^5^ < Re < 10^7^ (e.g., Cf = 0.0576Re^-1/5^, Cf = 0.0592Re^-1/5^, Cf = (2log10(Re)-0.65)^-2/3^, besides others) [25]. We recomputed our data for all approximations and compare it with the ITTC-57. The differences across all equations for the total drag and drag coefficient ranged between 1.25% and 2.22%. Therefore, it seems that there is a negligible difference according to the correlation model selected, at least for such young swimmers. It remains to be shown if the same happens in adult counterparts.

Linear regression models between experimental and analytical procedures showed a high adjustment for both passive drag and passive drag coefficient (Fig 1). The drag force in absolute units and drag coefficient after logarithmic transformation were the ones showing the best goodness-of-fit and lower error of the estimations. The proportional bias in the Bland-Altman plot for the drag force (absolute values) and drag coefficients (log transformation) is consistent with such models (Fig 1) and in accordance to what is reported in other settings.

If there is no displacement, the drag force acting upon the swimmer is null (Eq 1). If the model is adjusted to such condition, its goodness-of-fit increases furthermore (Eqs 14 and 15). As much as we understand, this procedure was reported at least a couple of times [8,26]. According to the cut-off values set for the qualitative and effect size analysis (please refer to the statistics subsection), both relations are very high (0.81 ≤ R^2^ < 1.0). Nevertheless, Eq 14 suggests that there is an overestimation (if y = m^.^x + c, c = 0 and m < 1) of the passive drag, and Eq 15 is an underestimation of the passive drag coefficient (if y = m^.^x + c, c = 0 and m > 1) calculating the hydrodynamics with the analytical procedures. On average, the difference between methods was -7.002N (95% CI: -40.480 to 26.475) for the passive drag and 0.127 (95% CI: 0.007 to 0.247) for the passive drag coefficient (Fig 1). An individual analysis shows that 10 of 60 subjects had clear underestimations of the passive drag force and the remaining ones a good-fit or obvious overestimations. The vast majority of the underestimations fall between the 0 and -10N difference and were clustered in a mean value of 40 to 90N. This is consistent to what depicts the log-log plot. Regarding the drag coefficient, all but a couple of subjects presented overestimations. Nevertheless, 22 are mostly clustered between 0 and 0.1 (dimensionless, in absolute units). Again, it is assumed that estimated data is valid if at least 80% of the plots are within the 95% confidence interval limits [18], which did happen. Thus, although there is a very strong relation between both procedures, a bias exists, and a correction factor must be applied to report accurate data. To the best understanding of the authors, no paper is found in the literature aiming to compare the passive drag measured with different procedures. However, a similar research design was retrieved comparing two techniques (velocity perturbation method vs. measuring-active drag system) to assess the active drag force [27]. Interestingly, the authors reached a conclusion similar to ours that both methods measure essentially the same phenomenon and that the bias can be explained by the different assumptions that the tests are based on.

The partial contribution of *D*_*f*_ and *D*_*pr*_ to total passive drag was 14.12 ± 9.33% and 85.88 ± 9.33%, respectively. If other friction correlation models are selected, notably equations suggested for the range 10^5^ < Re < 10^7^, the partial contribution of *D*_*f*_ decreases in 1% to 2%. Only a couple of papers are found in the literature about this relationship as it is very challenging to gather such insight. Numerical simulations solving the Reynolds Average Navier-Stokes equations (including the Standard k-epsilon turbulence model) that govern the equation of motion of a fluid are one way to learn about it [28]. The *D*_*pr-*_ *D*_*f*_ relation was reported as being 85% to 15% [15] and 75% to 25% [16]. In both papers, the model was assumed as having a null roughness, which may lead to an underestimation of the *D*_*f*_. Displacing at 2.2 m/s and neglecting the *D*_*w*_ effect, the *D*_*pr-*_ *D*_*f*_ relation was experimentally measured as being 65% to 35% wearing a waist-knee swimsuit and 71% to 29% in a conventional suit [8]. Interestingly, our data match the 85% to 15% reported by Marinho et al. [15].

That said, total drag force on the surface is the sum of the *D*_*f*_, *D*_*pr*_ and *D*_*w*_ (Eq 4). Hence, in such case, the partial contribution of *D*_*f*_ and *D*_*pr*_ may change because *D*_*w*_ becomes the major determinant [14]. The *D*_*f*,_, *D*_*pr*_, and *D*_*w*_ will show a linear (D_f_ = k^.^v), quadratic (D_pr_ = k^.^v^2^), and proportional to the fourth power (D_w_ = k^.^v^4^) of speed, respectively [8] (Fig 2). Having the same subject performing several trials only the speed would change and remaining terms are kept constant. In such event, the relationship would be clearly v^2^. However, in this research we have different subjects performing the trials at different speeds. This happens because the gliding decay technique must be performed after a maximal push-off start. Hence, not only the speed changes but also other terms in the model. This might be one of the explanations why Fig 2 depicts a mainly linear relationship for the friction drag rather than the expected theoretical v^2^ according to Eqs 9, 10 and 11.

The following can be addressed as main limitations: (i) for this kind of research, larger sample sizes are needed. Young talented swimmers rather than elite counterparts were assessed because it is very challenging to recruit so many adult/elite swimmers to be part of the study; (ii) it would be very interesting in a near future to compare the analytical procedure with numerical simulations; and (iii) gliding below the surface is not the same as gliding on the surface, because in the later one wave drag is a determinant factor. So a deeper insight about the partial contribution of the three components (*D*_*f*_, *D*_*pr*_ and *D*_*w*_) to both passive and active drags on the surface is also needed.

As a conclusion, there is a strong relationship between the passive drag and passive drag coefficient assessed with experimental and analytical procedures. The analytical method is a novel, feasible and valid way to gather insight about one’s passive drag during training and competition. Analytical methods can be selected not only to perform race analysis during official competitions. It can also be used to monitor the swimmer’s status on regular basis during training sessions, without disruptions or time-consuming procedures. The partial contribution of pressure and friction drag components to total drag is roughly 85–15%.

## Supporting Information

S1 Dataset Partial dataset used in this research. (XLSX)
