# Correction: A Comparison of Experimental and Analytical Procedures to Measure Passive Drag in Human Swimming

**DOI:** 10.1371/journal.pone.0177038

**Published:** 2017-05-01

**Authors:** Tiago M. Barbosa, Jorge E. Morais, Pedro Forte, Henrique Neiva, Nuno D. Garrido, Daniel A. Marinho

The following information is missing from the Funding section: This project was supported by the National Funds through FCT—Portuguese Foundation for Science and Technology (UID/DTP/04045/2013)—and the European Fund for regional development (FEDER) allocated by European Union through the COMPETE 2020 Programme (POCI-01-0145-FEDER-006969).
